# Health status of male steel workers at an electric arc furnace (EAF) in Trentino, Italy

**DOI:** 10.1186/s12995-016-0095-8

**Published:** 2016-02-20

**Authors:** Roberto Cappelletti, Marcello Ceppi, Justina Claudatus, Valerio Gennaro

**Affiliations:** International Society of Doctors for the Environment (ISDE Italy), via della Fioraia 17/19, 52100 Arezzo, Italy; Istituto di Ricovero e Cura a Carattere Scientifico (IRCCS) Azienda Ospedaliera Universitaria “San Martino” Istituto Nazionale per la Ricerca sul Cancro (IST), largo R. Benzi 10, 16132 Genoa, Italy

**Keywords:** Steel workers, Electric arc furnace (EAF), Diffuse emission, Injury, Diabetes, Hypertension, Cardiovascular disease, Foundry dust, Lung cancer, Rheumatoid arthritis

## Abstract

**Background:**

The aim of this retrospective cohort study was to determine if the workers of an Electric Arc Furnace (EAF), which recycles scrap, had higher mortality and morbidity due to possible exposure to pollutants at work. EAFs do not run on coke ovens. In EAFs 40 % of the particulate matter (PM) is made up of PM _2.5_. The foundry dust contained iron, aluminum, zinc, manganese, lead, chromium, nickel, cadmium, mercury, arsenic, polycyclic aromatic hydrocarbons (PAHs), polychlorinated biphenyls and dioxins.

**Methods:**

*Mortality study*: a cohort of 331 exposed workers (6731 person-years) was studied from 19/03/1979 to 31/12/2009 (mean follow up 20.7 years). The group of exposed workers was compared to the general population and to a small control group of 32 workers from the same company.

*Morbidity study*: rates of exemption from health fee for the seven major diseases of 235 exposed workers were compared to the rates of exemption in the Province of Trento.

**Results:**

*Mortality study*: an excess mortality was found in the exposed workers as compared to the *general population* (SMR 1.13; 95 % CI: 0.76–1.62; 29 deaths) and to the *internal group* (RR 2.34; 95 % CI: 0.39–95.7). The mortality rate was increased for *all tumours* (SMR 1.36; 95 % CI: 0.75–2.29; 14 cases), for lung cancer (SMR 3.35; 95 % CI 1.45–6.60; 8 cases), for *ischemic heart disease* (SMR 1.27; 95 % CI: 0.35–3.26; 4 cases), for *chronic liver disease* (SMR 1.16; 95 % CI: 0.14–4.20; 2 cases) and for *injury and poisoning* (SMR 1.32; 95 % CI: 0.48–2.88; 6 cases).

*Morbidity study*: there was a statistically significant increase of diabetes, rheumatoid arthritis, hypertension and cardiovascular diseases in exposed workers.

**Conclusions:**

With the limitations of this relatively small cohort, we found a statistically significant increase of diabetes, cardiovascular diseases and deaths due to lung cancer in exposed workers. These findings cannot be explained by PAH exposure alone; metal particulates are the most important pollutants in the working area of EAFs. A reliable method for measuring metal PM in tissues is urgently needed for exposure assessment. This study underlines the necessity to maximize the standards of security toward *foundry dusts/diffuse emission.* Further studies on EAF’s are needed to confirm our findings and to increase statistical power.

## Background

The correlation between increased rate of mortality for some tumours (mainly for lung cancer) and working in a steel foundry is well-known [1–13]. In 1987 the International Agency for Research on Cancer (IARC) classified iron and steel founding as being carcinogenic to humans (Group 1) [13]. There is still no agreement on which of the following agents (alone or in combination with) are responsible for the adverse effects on health in steel workers, i.e. polycyclic aromatic hydrocarbons (PAHs) and silica [2, 6, 11, 12], chromium and nickel [3, 9, 11, 12], iron dust and mixture of dusts [2], oil mist [2, 10], welding fumes [2, 11], particulate matter (PM) containing nickel and arsenic [14]. The Electric Arc Furnaces (EAFs) play an increasingly important role in modern steel production. Today the percentage of electric arc furnace steel of the overall steel production in the EU-27 is 41.8 % [15]. In Italy with 61 % and in Spain with 77 %, the production of EAF steel is significantly higher than steel production via the blast furnace/basic oxygen furnace route [15]. In Italy there are 35 EAFs which employ 40,000 adepts. EAFs do not run on coke ovens. A coke oven produces, benzo-[a]-pyrene, benzene, coke dust and other PAHs, which may cause lung tumours [16–18]. These facts may generate the misleading idea that EAFs, not been equipped with a coke oven, are less hazardous to health. In iron foundries the proportion of ultrafine particle is considerably higher as compared to outdoor ambient air [19]. In EAFs, 40 % of the particulate matter (PM) is made up of PM_2.5_ [20]. Ample data has been published on the damage caused by PM_2.5_ and ultrafine PM (PM _<0.1_) in humans [21]. PM_2.5_ has been recently classified by IARC as being carcinogenic for humans (Group 1) [22]. Metal particulate matter poses additional risks [23–25]: it can persist over a long period of time both in the environment and in the body tissues [26, 27]. Metal content enhances oxidative stress and promotes the release of pro-inflammatory mediators [24]. Evidence of increased DNA damage and lipid peroxidation through oxidative stress has been found in foundry workers [28]. Long term exposure of steel workers to air particles containing nickel or arsenic can induce histone modification as an epigenetic mechanism leading to cancer [14]. In EAFs there is also an exposure to polychlorinated-biphenyls (PCBs) and polychlorinated dibenzo-p-dioxin/dibenzofurans (PCDD/Fs) [29, 30]. As of yet no epidemiological occupational studies have been carried out in EAFs. This type of steel foundry could represent an ideal model for studying the effect on health of the metal particulate matter. In the present study we report the data of mortality and morbidity of an EAF located in an alpine valley in the municipality of Borgo Valsugana (Italy). We tested the hypothesis that the various substances in Table 1, released as fine and ultrafine PM through *diffuse emissions*, could have produced adverse health effects in the workers. By definition *diffuse emissions* occur during regular operations, as opposed to *fugitive emissions* that happen during irregular operations [15].

**Table 1:** Composition of foundry dust^a^ and corresponding toxicological data

Elements	mg/kg of dusts	% of total	IARC Classification^b^	non-cancer health effects
Iron (Fe)	197000	78		Intestine, liver, heart
Aluminium (Al)	18970	7.64		Central Nervous System (CNS), bone
Zinc (Zn)	14340	5.68		Intestine, genitourinary, CNS [67, 73]
Manganese (Mn)	12500	4.95		CNS [74]
Total hydrocarbons	2960	1.17	(see P.A.H.)	(see P.A.H.)
Lead (Pb)	2860	1.13	2A (inorganic compounds) and 2B	CNS, liver, kidney [75]
Chromium (Cr) (total)	2230	0.88	1	Skin, bone, kidney [75]
Copper (Cu)	810	0.32		Liver, kidney, skin, bone, teeth
Nickel (Ni)	510	0.20	1 (compounds) and 2B	Skin, bone, teeth, diabetes [25, 56]
Cadmium (Cd)	30	0.0118	1	Liver, kidney, eye, bone, diabetes [57]
P.A.H.	3.66–44.67	0.009	1 (benzo[a]pyrene), 2A, 2B and 3	Heart (attack), teratogenic [16]
Mercury (Hg)	9	0.0035	2B (methyl mercury) and 3	CNS, liver, kidney, diabetes [56]
Arsenic (As)	4	0.0015	1	CNS, diabetes [56]
Polychlorinated biphenyls (PCB)	0.092–0.693	0.0001	1	Endocrine disruptor, teratogenic
Dioxins (PCDD/F)	107.8 ng/TEQ	/	1	Endocrine disruptor, teratogenic [44]

^a^from Agenzia Provinciale per la Protezione dell’Ambiente (APPA) of Trento, 12^th^ December 2006 (dioxins excluded)

^b^International Agency for Research on Cancer (IARC) classification: group 1: carcinogenic for humans; group 2A: probable carcinogenic for humans; group 2B: possible carcinogenic for humans; Group 3: unclassified as to carcinogenic for humans

### EAF characteristics

The EAF of Borgo Valsugana, in the Province of Trento (Trentino), Italy, produces roughly 550,000 tons/year (110 tons/h) of semi-finished products (steel billets) from scrap recycling. At the beginning of the activity in 1979, when working only on a 4^Th^ hole extraction, up to 80 Kg of dust per hour were estimated to be released into the environment as *diffuse emissions*. There have been several technical interventions throughout the years and a doghouse and canopy hoods were installed. In 2011 total quantities of *diffuse emissions* were evaluated to be 2–5 kg/h of dust, as compared to 0.2–0.6 kg/h present in the channelled emissions [31]. Emissions from this steel foundry were found to be present in a perimeter of 2 km around the foundry, containing PCDD/F and PCB [32]. *Diffuse emissions* in EAFs arise mainly from the charging of the oven and can be minimized by isolating the oven area and by capturing them and transferring them into ducted emissions for further treatment.

## Methods

The company record books, which had been confiscated by the General Prosecutor of Trento during an inquiry about pollution, were utilized to study the workers. 20 % of the workers were hired via temp agency, and therefore it was impossible to study this group of workers. Dimension of the cohort, mean duration of employment, person-years, average time of follow-up, age at entry, and drop outs are summarized in Table 2. Seventeen workers, registered as “technical employee”, were excluded. The status of life (alive or dead) was certified by the respective municipality where each worker resided, and partially by directly verifying medical records. Causes of death were obtained, when possible, from the death certificate of the National Institute of Statistics (ISTAT). Otherwise, the Nominal Registry for the causes of death (2005–2009) at the Provincial Health Agency (APSS) in Trento and the medical records found at the Hospital of Borgo Valsugana were utilized. In the absence of medical records, three cases of death were not included in the specific causes of death, although there were testimonies which we judged trustworthy (one death possibly due to myocardial infarction, one to a car accident and one to carbon monoxide poisoning). Steel production in Borgo Valsugana began on 19/03/1979, when this study was started. Subjects were enrolled to the study on the day they were employed. The follow-up began on the date of employment (not before 19^th^ march 1979) and terminated either with the death or on 31^st^ of December 2009. Status of life was verified. A worker was classified as “being exposed” after having worked at the foundry for at least one year.

**Table 2:** Principal characteristics of the foundry worker cohort

Cohort foundry workers			
	Workers ≥ 1 year (exposed)	Control group (office work)	Workers < 1 year
Workers studied	331	32	111
Mean duration of employment (median)	8.6 (5.5) years	5.6 (3.1) years	<1 year
Lost to the follow-up	22 (6 %)	15 (31 %)	16 (12 %)
Person-years	6731.0	491.3	2387.1
Average duration of the follow-up (range)	20.5 (1.2–30.8) years	15.5 (0.2–30.8) years	21.0 (2.2–30.8) years
Mean age at entry	31.1 years	35.5 years	29.2 years
Observed deaths (n.)	29	1	7
Not included	17 (exposition not certain) + 3 females	12 females	
TOTAL	373	59	127

### Analysis of mortality

331 male “workers” were enrolled to the study. 32 male subjects, whose life status was known, were chosen as controls (clerks, managers and a watchman). Women were excluded from the study. The high percentage of drop outs in the control group (30.4 %) could be explained by the high mobility of these subjects, making it difficult to trace them. The person-years were calculated by age groups for periods of five years from 1979 to 2009 (6 groups). *Expected deaths* for *all causes* and for each specific disease were evaluated by utilizing the rate of mortality in the Region Trentino-Alto Adige [33]. Every five years the specific rate of mortality was applied to each age group, using an arithmetic average of the yearly rates. Be it that the years 2004 and 2005 were lacking, the average depends on the years available. Standardized Mortality Ratios (SMR), 95 % Confidence Interval (95 % CI) and *p* values (p) were calculated for the main pathologies and causes of death. STATA software (Statacorp 2009) was utilized for the calculations [34]. Other factors, beside the occupational risk factors, were analysed, when present in the medical records, like smoking and alcohol consumption.

### Analysis of morbidity

Exemption from health fees in the group of workers employed for more than one year was adopted as a surrogate for the prevalence of disease and compared to the rate observed in the Province of Trento*.* The analysis was limited to seven exemptions, that accounted for more than 2/3 of the total exemptions in the province of Trento, i.e. patients suffering from malignant tumours (code 048); hypertension with organ damage (“complicated” hypertension is used in this study as synonym: code 0031); hypertension (without any organ damage: code 0A31); diabetes mellitus (code 013); cardiopulmonary vascular diseases (cardiovascular disease is used as synonym: code 0A02); rheumatoid arthritis (code 006) and asthma (code 007). The status of exemption from health fees for the given pathology for 299 residents in the Province of Trento up to the 28^th^ of January 2011 was obtained from the APSS of Trento. 235/299 male subjects (workers and clerks), having one or more than one year of employment at the steel foundry, were studied. The workers studied for health fee exemptions were less than those studied for mortality, because workers not residing in the Province of Trento were excluded, and residents in some municipalities were accidentally excluded in the formulation of the request of data to APSS. The census of the provincial population was taken in January 2011. The rates of exemption registered in the Province of Trento were calculated by dividing the total number of exemption for a certain disease by the number of males over 20 years of age reported for on the 1^st^ of January 2011. We utilized the Mantel-Haenszel method to adjust the result for age; three age groups were utilized: 20–64, 65–74, 75 and over. 17 out of 64 non-exposed workers, exempt from health fees, were inserted into an additional cohort for an internal comparison. The remaining 47 were excluded since they did not fulfil the criteria of this study: 4 women, 34 workers employed under one year; and 9 technical employees.

## Results

### Analysis of mortality

Table 3 shows the observed and expected number of deaths of the foundry workers for *all causes* and for the main groups of diseases (cause-specific mortality). The SMR for all causes was 1.13 (95 % CI:0.76–1.62), when the general male population was used as the reference. Average age at death was 54 years. There was an increase of *all cancers* (SMR 1.36; 95 % CI: 0.75–2.29; 14 cases), that was statistically significant for lung cancer (SMR 3.35; 95 % CI: 1.45–6.6; 8 cases). With the exception of the 8 cases of lung cancer, the other deaths due to malignant tumours were localized in 6 different sites (oesophagus, stomach, colon-rectum, pancreas, testicle and multiple myeloma). There was also a non-statistically significant increase for *ischemic cardiac diseases* (SMR 1.27; 95 % CI: 0.35–3.26; 4 cases) *chronic liver diseases* (SMR 1.16; 95 % CI: 0.14–4.2; 2 cases), *injuries and poisoning* (SMR 1.32; 95 % CI: 0.48–2.88; 6 cases). When computing the overall mortality SMR (all causes) for different time lengths of employment (1–4; 5–9; ≥10) there was an inverse relationship (Table 4), indicating a strong *healthy survivor effect* [35]. This cohort is characterised by a high turn-over of the personnel: 25 % of the total cohort of blue-collar workers was employed less than 1 year, 55 % less than 5 years. There were no lung cancers before 3 years of exposure. In workers who died from lung cancer, the mean age at death was 63.7 years, while the mean age at death from lung cancer in the Province of Trento was 72.1 years (2003–2006). Seven out of eight deaths from lung cancer occurred between 2005 and 2009. SMRs by exposure duration and by time since first exposure for all tumours and lung cancer are synthetized in Tables 4 and 5. In 91 workers employed at present, the habit of smoking was reported only for 17 workers (18.7 %). As compared to the smoking habit of the general population in the Province of Trento (29 % smokers; 29.2 % ex-smokers) [36], there was a 12 % excess of smokers in this small sample of workers. Regarding alcohol consumption, 2 workers declared that they consumed a moderate quantity of wine daily, whereas 11 denied alcohol intake. In 8 workers, who died from lung cancer, only in 6 did we obtain information on their smoking habit: 1 was not a smoker, 2 were ex-smokers and 3 had been smokers (20 cigarettes/per day). Six exposed workers died because of *injuries or poisoning*: 4 being fortuitous non work-related traumata, one death due to work-related trauma and one due to suicide. Three of these deaths occurred while still being employed, whereas three occurred successively. Mean length of employment time was 10.6 years (range 2–17 years). 331 exposed workers were confronted with 32 white collar workers of the same company. In this *internal* confrontation the Relative Risk ratio (RR) was 2.34 (95 % CI: 0.39–95.72). We calculated the avoidable deaths to be 16.6 (95 % CI: 0–28.7). Finally, we studied the workers with less than one year of employment separately. The SMR for all causes was 0.97 (95 % CI 0.39–2.0). Seven workers, employed under one year, died as follows: four cases of *injury and poisoning* (SMR: 2,56; 95 % CI: 0.8–6.1; 3 suicides, 1 car accident), 1 case of gastrointestinal haemorrhage (liver cirrhosis for alcohol abuse), 2 cases due to cancer (SMR 0.75; 95 % CI: 0.09–2.71; 1 cancer of the larynx, 1 pancreatic cancer) (see also Table 4).

**Table 3:** Mortality of workers exposed ≥ 1 year (reference: general population)

ICD 9^a^	ICD 10	Cause of death	Observed	Expected	SMR	95 % CI	*P*
001 - 999	A00–T98	**All causes**	29^b^	25.69	1.13	0.76–1.62	0.527
140 - 239	C00–D48	**All tumours**	14	10.27	1.36	0.75–2.29	0.283
162	C33–C34	**Malignant tumors of the larynx**, **trachea, bronchi, lungs**	8	2.39	**3.35**	**1.45–6.60**	**0.010**
410 - 414	I20–I25	**Ischemic heart disease**	4	3.14	1.27	0.35–3.26	0.768
571	K70; K73–K74	**Chronic liver disease**	2	1.72	1.16	0.14–4.20	0.837
800 - 999	S00–T99	**Injury and poisoning**	6	4.53	1.32	0.48–2.88	0.604

^a^Till 2002. ^b^In the absence of medical records, 3 cases were not included in the specific causes of deaths. CI = Confidence Interval

In bold, statistically significant value

**Table 4:** SMRs by years of exposure (employment) for selected causes of death

ICD 9	Cause of death	Years of exposure											
		0 - < 1 year			1–<5 years			5–<10 years			10 + years		
		Obs	SMR	95 % CI	Obs	SMR	95 % CI	Obs	SMR	95 % CI	Obs	SMR	95 % CI
001–999	All causes	7	0.97	0.39–2.00	11	1.29	0.64–2.31	9	1.21	0.55–2.30	9	0.94	0.43–1.79
140–239	All tumours	2	0.75	0.09–2.71	5	1.68	0.54–3.92	4	1.32	0.36–3.38	5	1.17	0.38–2.73
162	Malignant tumors of the larynx, trachea, bronchi, lungs	1	1.66	0.04–9.25	3	4.35	0.90–12.71	3	4.11	0.85–12.01	2	2.06	0.25–7.45

*Obs* observed death, *Exp* expected death, *CI* confidence interval

**Table 5:** SMRs by time since first exposure for selected causes of death (workers exposed ≥ 1 year)

ICD 9	Cause of death	Years since first exposure											
		1–<10 years				10–<20 years				20 + years			
		Obs	Exp	SMR	95 % CI	Obs	Exp	SMR	95 % CI	Obs	Exp	SMR	95 % CI
001–999	All causes	8	6.18	1.29	0.56–2.55	7	8.03	0.87	0.35–1.80	14	11.48	1.22	0.67–2.05
140–239	All tumours	2	1.59	1.26	0.15–4.54	4	3.11	1.29	0.35–3.29	8	5.56	1.44	0.62–2.84
162	Malignant tumors of the larynx, trachea, bronchi, lungs	1	0.36	2.78	0.07–15.48	1	0.75	1.33	0.03–7.43	6	1.27	4.72	1.73–10.28

### Analysis of morbidity

88 out of 235 workers had 118 health fee exemptions (50.2 %). The rate of exemption for all diseases in the Province of Trento is about 28 % for the male sex. Table 6 summarizes the rates of health fee exemption of exposed workers as compared to the rate exemption found in the Province of Trento. The rate of exemption in exposed workers was statistically significant for diabetes (RR = 2.24; *p* = 0.0002), hypertension (RR = 2.01; *p* = 0.0018), and rheumatoid arthritis (RR = 6.18; *p* = 0.013). After adjustment for age (Mantel Haenszel method), the increased rate of cardiovascular diseases also became statistically significant. As foreseen, by *internally comparing* the workers, less fee exemptions were requested among the non-exposed group: in fact, there were only two cases (048 and 0A31) versus the expected 8.5. The exemption 048 (malignant tumour) was referred to a night watchman.

**Table 6:** Health fees exemptions of 235 workers^a^ compared to the exemptions of the provincial population

	Workers (*n* = 235)	Province of Trento (PT) (n. of male population ≥ 20 years = 203332)			PT, Adjusted for age (Mantel Haenszel)	
Diseases	n. of cases	n. of cases	RR	P	RR	95 % CI
Tumours	12	9655	1.08	0.7580	1.19	0.69–2.04
Cardiovascular Diseases	5	8676	1.50	0.1067	**1.74**	1.07–2.82
Diabetes	25	9630	**2.24**	**0.0002**	**2.39**	1.67–3.41
Rheumatoid Arthritis	3	420	**6.18**	**0.0133**	**6.17**	2.00–19.02
Asthma	2	1968	0.88	1.0000	1.08	0.27–4.31
Non-complicated Hypertension	30	11655	**2.23**	**0.0001**	**2.44**	1.75–3.40
Complicated Hypertension	14	6033	**2.01**	**0.0181**	**2.22**	1.35–3.65

^a^male sex, exposed ≥ 1 year

In bold statistically significant values. “Cases” refer to health fees exemptions

## Discussion

### Limitations

EAFs are small plants as compared to integrated foundries which include all the production phases starting from the initial processing of the iron ore. EAFs use scrap as raw material and in the case of Borgo Valsugana only semi-finished products are obtained. Therefore, this cohort is relatively smaller as compared to other studies on foundries and may have a limited statistical power. We did not have access to a similar *external* comparison *group* of workers to minimize the *healthy worker effect* (HWE) [35, 37–42]. Instead, we compared mortality and morbidity of 331 exposed workers (≥1 year) with the *general population* of the Region Trentino Alto Adige (Table 3) and also with a small group of clerks working at the same foundry (Table 7). Although this internal comparison was numerically limited, the relative risk ratio between exposed workers/non-exposed clerks in our study was well above the unity (RR 2.34), in line with a previous study [2]. We could not differentiate among the different type of work tasks, because no data were available in the Registration Book. In this factory the working environment was constituted by a single non-divided shed, so that all workers were exposed to the same pollutant/s. Some tasks (e.g. continuous casting, furnace workers) could have had a higher level of exposure than others (e.g. maintainers, operators), but in this small plant it was not infrequent to go from a type of task to another. Besides age, other confounders, like diet and the socio-economical level were not considered in this study. On the other hand, having studied both mortality and morbidity has the advantage of a more comprehensive evaluation of the problem, bearing in mind the pyramid phenomenon of effects on health.

**Table 7:** Internal comparison between workers exposed ≥ 1 year and the non-exposed workers (white collars)

Groups (N. of subjects)	Observed	Expected	SMR	95 % CI	Person Years
Exposed Workers ≥ 1 year (n.331)	29	25.69	1.13	0.76–1.62	6731.0
Non-exposed (controls) (n.32)	1	2.09	0.47	0.01–2.67	491.3
Comparison exposed/non exposed workers			RR: 2.34	0.39–95.72	
Avoidable deaths			16.6	0–28.7	

### Healthy worker effect

The lower SMR of workers as compared to the general population is due to the so-called *healthy worker effect* (HWE), arising largely from employment selection factors [35, 37–42]. In the absence of workplace hazards, all cause SMRs for Caucasian men is 0.70–0.85 [9]. In this study we found a SMR of 1.13 (95 % CI: 0.76–1.62), which is similar to what has been found by others [5, 7, 8]. We attributed the inverse dose–response relationship for *all causes* (Table 4) to a *healthy survivor effect*, a component of the *healthy worker effect* [35]. The *healthy survivor effect* is attributed to a strong selection throughout the years due to an early dropping out from work by those workers who are feeble [35, 37–42].

### Mortality for lung cancer

Our data confirm what has been already reported in literature: there is a high statistically significant increase (*p* = 0.01) of deaths due to lung cancer (8 cases observed versus the expected 2.39). In this study there are limited data regarding the habit of smoking and alcohol consumption amongst the workers. However, it does not seem that differences in life style could justify the high rate of mortality for lung cancer. The precocious median age at death for lung cancer in the exposed workers (minus 8.4 years) as compared to the Province of Trento suggests the presence of carcinogenic factors other than cigarette smoking. The relationship between environmental pollution due to particulate matter and lung cancer is well-known [43].

### Gradient effect

In this study no dose–response relationship was found for tumours when considering the duration of employment. On the other hand, when considering the time since the first exposure, there was an increase of SMRs for all tumours and lung cancer after 20 years (Table 5). While an increasing risk with employment duration supports causal inference, the absence of an increasing risk with employment duration is not a strong evidence against it [9, 37]. In mortality studies of steel workers the absence of a gradient effect for cancer deaths is rather frequent: among 7 studies, that reported dose–response assessment [1, 3–7, 9], 3 studies found no dose–response relationship [1, 6, 9], while 4 studies found a certain dose–response relationship [3–5, 7]. There can be several explanations for this. First, the *healthy survivor effect* should be taken into consideration. Secondly, there is a *competition between diseases* so that those who die, e.g. from cardiovascular disease or injuries, have no time to develop cancer. Thirdly, the length of employment does not always correlate with the level of exposure. In the case of steel workers, urine or serum analysis of metals do not always correlate with metal PM content in tissue, so a reliable method for measuring fine and ultrafine metal PM in tissue is urgently needed [26]. Finally, the difficulty of evidencing a dose–response relationship imply that the substances in question have their characteristic way of reacting. A given substance may accumulate in the tissues over time and induce a cancer slowly throughout life [44].

### Injuries

Though not statistically significant, we found an excess (+32 %) of injuries. Increase of mortality for injuries is frequently reported in steel worker studies [2, 5, 9, 45]. Barreto et al. observed in Brazil an increase of mortality due to car accidents [45] and suggested that work-related factors could be rotating shift work, high levels of noise and mental illness, which is “a major health problem in steel workers” [45]. The Chinese study found that increased mortality for injuries paralleled increased mortality for all causes when certain types of exposures are concerned (iron, welding fumes, coal, asbestos, heat, PAHs and benzene), but not for other types of exposures (silica, wood and CO) [2]. Neurobehavioral function alterations have been documented in steel workers [46, 47]. Recent animal studies on neurological toxicity of metal particulates on a nanometric scale have shown that these particles have a high tropism for the brain [48]: they can cause an alteration of the sensorial and cognitive functions, damage to the blood–brain barrier, the neural cells, the glial cells and myelin. Finally, it is recognized that subjects exposed to nanoparticulates may have an altered function of their mental health [48–50]. Therefore, we consider it possible that high levels of metal particulates and neurotoxic substances (Table 1) could cause subtle effects on the capability of reaction and attention and could explain the increased number of accidents in steel workers. Further studies are needed.

### Morbidity

Our study shows a statistical significant increase of diabetes, rheumatoid arthritis, hypertension and cardiovascular diseases in exposed workers as compared to the population of the province (Table 4). We used the exemption of health fees as a surrogate for the prevalence of diseases. As is known, the analysis of the exemption from health fees may give results, which underestimate the real prevalence of diseases. We assumed that, when internal comparisons are made, the rate of exemption from health fees is a good indicator of health status of a given population in the absence of other data. The validity of this assumption, is confirmed by the results obtained from the internal analysis of the health fee exempted cohort, comparing exposed workers to *non-exposed* workers: there were only two applications for exemption from health fees in the small group of controls. Age was shown not to be a confounding element. At present in the Italian Health System, who suffers from a chronic disease may request the exemption from health fees, independently from his/her socio-economic status. The seven diseases considered in this study account for more than 2/3 of the total number of exemptions for diseases in the Province of Trento, so we consider them relevant in assessing a health status. There is no evidence that this cohort of steel workers had better access to health care than the general population. We confirm what Martinez et al. [51] found in Brazil, i.e. a high prevalence of diabetes and hypertension in foundry workers: the prevalence of diabetes in Brazil was 11.5 %, and we observed a prevalence of 10.6 % in the foundry workers of Borgo Valsugana. Martinez et al. also reported more cases of diabetes in the exposed workers as compared to clerks. Diabetes is the most frequent chronic disease in industrialized countries and it is now increasingly evident that it is also associated with environmental toxic agents like heavy metals and PCDD/Fs [52–57]. Moreover, the relationship between hypertension/cardiovascular diseases and air pollutants is well known [58–67].

### Diffuse emissions/foundry dust

This study shows a notable deterioration of the health status of the exposed workers, as compared to the referral groups. This negative finding could be due to the working conditions. We do not have detailed information on PM concentration and metal content of the air within the foundry, although we assume that it has a similar composition and proportion of the elements found in the dust which had deposited (Table 1). The level of PNOC (particulates not otherwise classified) might have been quite high, sometimes exceeding the limits of 10 mg/Nm^3^ established by the ACGIH. In 2007, the local Environment Protection Agency (APPA) reported that the quantity of diffuse emissions during charging of the electric arc furnace of Borgo Valsugana was *“some grams per normal cubic meter and could be perceived by the naked eye”*. The working conditions in this EAF have improved after the instalment of new canopy hoods and with the automation of several working processes. The danger of foundry dusts inhaled through diffuse emission is inherent to the deposition of metal PM in body tissue and to the release of toxic and carcinogenic metals. Common steel can release nickel and chromium in body tissues by corrosion. Regarding fine and ultrafine metal PM there is a tremendous lack of knowledge concerning tissue distribution, clearance (half-life) and toxic health effects. Data on PM level and composition in the air of steel foundries, as well as the health risk assessment on metal exposure, are available from other studies [14, 28, 68, 69]. In fact, Angelo Borroni (Polytechnic University of Milan) studied over 400 samples of air taken within the working areas of 10 Italian EAFs from 1985–2005; in the *oven working area* the levels of PNOC, manganese, lead, silica and calcium oxide were not infrequently (roughly 1/8) above the limits determined by the ACGIH (silica is no longer utilized as a refractory material since 1990) (unpublished data). Furthermore, Liu et al. reported that the health risk assessment on metal exposure in foundries showed that the cancer risk for cadmium, chromium and nickel were all above 1 × 10 (−6) [28]. To avoid underestimation of health risk assessment we suggest to consider steel “*foundry dusts*” *in toto*, as the sum of the individual risks can neglect the synergistic effect of its single pollutants. Foundry dust should be considered a “*high hazard*” pollutant. PAH exposure alone cannot explain the excess of diabetes in our study, that may better correlate with heavy metals and PCDD/F. Exposure to PAH in EAFs has been reported to be in the median range as compared to other types of industries [70]. Therefore, PAH exposure does not seem to be so relevant in EAFs as compared to other substances present in the foundry dust, i.e. fine and ultrafine metal PM deriving from high temperature fusion processes. To limit the diffuse emissions of the EAFs, which contain dangerous micro-pollutants like PCCD/F, the European Commission has underlined the necessity of total enclosure buildings [15]. On the other hand, nowadays the EU regulation allows for over 98 % cut down of the total dust produced by the oven. This means that up to 27 kg/h of dust could be released into the environment, when roughly 1360 kg of dust per hour is produced as is the case in Borgo Valsugana. Unfortunately, as of yet, in Europe there are still EAFs plants which work only with a fourth hole extraction [15].

## Conclusions

With the limitations of this relatively small cohort (331 exposed workers, 6731 person-years) and the lack of a consistent comparison group of workers, this study shows a significant increased risk of lung tumours, diabetes, rheumatoid arthritis, hypertension and cardiovascular diseases as compared to the general population. This outcome may be attributed to working conditions in the EAF, where the numerous toxic and carcinogenic substances contained in the *foundry dust/diffuse emissions* represent the most important risk factors for quantity and biological plausibility. PAHs alone cannot explain the increased prevalence of diabetes. A reliable method for measuring metal PM in tissue is urgently needed for a better exposure assessment in future studies. Our data underline the importance to maximize the standards of security for *foundry dust/diffuse emissions* in EAF, so as to protect *in primis* the workers and then the population living around this foundries [71, 72]. Further studies on EAF’s are needed to confirm our findings and to increase statistical power with pooled analyses.
